# Supplementary material for: HIVprotI: an integrated web based platform for prediction and design of HIV proteins inhibitors
Source: J Cheminform. 2018 Mar 9;10:12. doi: 10.1186/s13321-018-0266-y (PMC5845081; doi:10.1186/s13321-018-0266-y)
Supplement: Supplementary file 2 — Additional file 2. Source code of HIVProtI web server. [file 13321_2018_266_MOESM2_ESM.zip › HIVprotI_Source-code/hivprotI/weka-3-6-6/doc/weka/gui/SetInstancesPanel.html]

SetInstancesPanel


---


|  |  |  |
| --- | --- | --- |
| **Overview** \| **Package** \| **Class** \| **Tree** \| **Deprecated** \| **Index** \| **Help** \| **Weka's home** | |  |
| **PREV CLASS**   **NEXT CLASS** | **FRAMES**    **NO FRAMES**     **All Classes** |  |
| SUMMARY: NESTED \| FIELD \| CONSTR \| METHOD | DETAIL: FIELD \| CONSTR \| METHOD |  |


---


## weka.gui Class SetInstancesPanel

```
java.lang.Object
  java.awt.Component
      java.awt.Container
          javax.swing.JComponent
              javax.swing.JPanel
                  weka.gui.SetInstancesPanel
```

**All Implemented Interfaces:**: java.awt.image.ImageObserver, java.awt.MenuContainer, java.io.Serializable, javax.accessibility.Accessible

---

``` public class SetInstancesPanel extends javax.swing.JPanel ```

A panel that displays an instance summary for a set of instances and
lets the user open a set of instances from either a file or URL.
Instances may be obtained either in a batch or incremental fashion.
If incremental reading is used, then
the client should obtain the Loader object (by calling
getLoader()) and read the instances one at a time. If
batch loading is used, then SetInstancesPanel will load
the data into memory inside of a separate thread and notify
the client when the operation is complete. The client can
then retrieve the instances by calling getInstances().

**Version:**
:   $Revision: 7059 $

**Author:**
:   Len Trigg (trigg@cs.waikato.ac.nz)

**See Also:**: Serialized Form

---

**Nested Class Summary**

**Nested classes/interfaces inherited from class javax.swing.JComponent**

`javax.swing.JComponent.AccessibleJComponent`

**Nested classes/interfaces inherited from class java.awt.Component**

`java.awt.Component.BaselineResizeBehavior`


**Field Summary**

**Fields inherited from class javax.swing.JComponent**

`TOOL_TIP_TEXT_KEY, UNDEFINED_CONDITION, WHEN_ANCESTOR_OF_FOCUSED_COMPONENT, WHEN_FOCUSED, WHEN_IN_FOCUSED_WINDOW`

**Fields inherited from class java.awt.Component**

`BOTTOM_ALIGNMENT, CENTER_ALIGNMENT, LEFT_ALIGNMENT, RIGHT_ALIGNMENT, TOP_ALIGNMENT`

**Fields inherited from interface java.awt.image.ImageObserver**

`ABORT, ALLBITS, ERROR, FRAMEBITS, HEIGHT, PROPERTIES, SOMEBITS, WIDTH`


**Constructor Summary**

`SetInstancesPanel()`
  
          Create the panel.


| **Method Summary** | |
| --- | --- |
| `void` | `addPropertyChangeListener(java.beans.PropertyChangeListener l)`             Adds a PropertyChangeListener who will be notified of value changes. |
| `void` | `closeFrame()`             closes the frame, i.e., the visibility is set to false |
| `Instances` | `getInstances()`             Gets the set of instances currently held by the panel |
| `Loader` | `getLoader()`             Gets the currently used Loader |
| `javax.swing.JFrame` | `getParentFrame()`             Returns the current frame the panel knows of, that it resides in. |
| `boolean` | `getReadIncrementally()`             Gets whether instances are to be read incrementally or not |
| `InstancesSummaryPanel` | `getSummary()`             Gets the instances summary panel associated with this panel |
| `void` | `removePropertyChangeListener(java.beans.PropertyChangeListener l)`             Removes a PropertyChangeListener. |
| `void` | `setInstances(Instances i)`             Updates the set of instances that is currently held by the panel |
| `void` | `setInstancesFromFileQ()`             Queries the user for a file to load instances from, then loads the instances in a background process. |
| `void` | `setInstancesFromURLQ()`             Queries the user for a URL to load instances from, then loads the instances in a background process. |
| `void` | `setParentFrame(javax.swing.JFrame parent)`             Sets the frame, this panel resides in. |
| `void` | `setReadIncrementally(boolean incremental)`             Sets whether or not instances should be read incrementally by the Loader. |

**Methods inherited from class javax.swing.JPanel**

`getAccessibleContext, getUI, getUIClassID, setUI, updateUI`

**Methods inherited from class javax.swing.JComponent**

`addAncestorListener, addNotify, addVetoableChangeListener, computeVisibleRect, contains, createToolTip, disable, enable, firePropertyChange, firePropertyChange, firePropertyChange, getActionForKeyStroke, getActionMap, getAlignmentX, getAlignmentY, getAncestorListeners, getAutoscrolls, getBaseline, getBaselineResizeBehavior, getBorder, getBounds, getClientProperty, getComponentPopupMenu, getConditionForKeyStroke, getDebugGraphicsOptions, getDefaultLocale, getFontMetrics, getGraphics, getHeight, getInheritsPopupMenu, getInputMap, getInputMap, getInputVerifier, getInsets, getInsets, getListeners, getLocation, getMaximumSize, getMinimumSize, getNextFocusableComponent, getPopupLocation, getPreferredSize, getRegisteredKeyStrokes, getRootPane, getSize, getToolTipLocation, getToolTipText, getToolTipText, getTopLevelAncestor, getTransferHandler, getVerifyInputWhenFocusTarget, getVetoableChangeListeners, getVisibleRect, getWidth, getX, getY, grabFocus, isDoubleBuffered, isLightweightComponent, isManagingFocus, isOpaque, isOptimizedDrawingEnabled, isPaintingForPrint, isPaintingTile, isRequestFocusEnabled, isValidateRoot, paint, paintImmediately, paintImmediately, print, printAll, putClientProperty, registerKeyboardAction, registerKeyboardAction, removeAncestorListener, removeNotify, removeVetoableChangeListener, repaint, repaint, requestDefaultFocus, requestFocus, requestFocus, requestFocusInWindow, resetKeyboardActions, reshape, revalidate, scrollRectToVisible, setActionMap, setAlignmentX, setAlignmentY, setAutoscrolls, setBackground, setBorder, setComponentPopupMenu, setDebugGraphicsOptions, setDefaultLocale, setDoubleBuffered, setEnabled, setFocusTraversalKeys, setFont, setForeground, setInheritsPopupMenu, setInputMap, setInputVerifier, setMaximumSize, setMinimumSize, setNextFocusableComponent, setOpaque, setPreferredSize, setRequestFocusEnabled, setToolTipText, setTransferHandler, setVerifyInputWhenFocusTarget, setVisible, unregisterKeyboardAction, update`

**Methods inherited from class java.awt.Container**

`add, add, add, add, add, addContainerListener, addPropertyChangeListener, applyComponentOrientation, areFocusTraversalKeysSet, countComponents, deliverEvent, doLayout, findComponentAt, findComponentAt, getComponent, getComponentAt, getComponentAt, getComponentCount, getComponents, getComponentZOrder, getContainerListeners, getFocusTraversalKeys, getFocusTraversalPolicy, getLayout, getMousePosition, insets, invalidate, isAncestorOf, isFocusCycleRoot, isFocusCycleRoot, isFocusTraversalPolicyProvider, isFocusTraversalPolicySet, layout, list, list, locate, minimumSize, paintComponents, preferredSize, printComponents, remove, remove, removeAll, removeContainerListener, setComponentZOrder, setFocusCycleRoot, setFocusTraversalPolicy, setFocusTraversalPolicyProvider, setLayout, transferFocusBackward, transferFocusDownCycle, validate`

**Methods inherited from class java.awt.Component**

`action, add, addComponentListener, addFocusListener, addHierarchyBoundsListener, addHierarchyListener, addInputMethodListener, addKeyListener, addMouseListener, addMouseMotionListener, addMouseWheelListener, bounds, checkImage, checkImage, contains, createImage, createImage, createVolatileImage, createVolatileImage, dispatchEvent, enable, enableInputMethods, firePropertyChange, firePropertyChange, firePropertyChange, firePropertyChange, firePropertyChange, getBackground, getBounds, getColorModel, getComponentListeners, getComponentOrientation, getCursor, getDropTarget, getFocusCycleRootAncestor, getFocusListeners, getFocusTraversalKeysEnabled, getFont, getForeground, getGraphicsConfiguration, getHierarchyBoundsListeners, getHierarchyListeners, getIgnoreRepaint, getInputContext, getInputMethodListeners, getInputMethodRequests, getKeyListeners, getLocale, getLocation, getLocationOnScreen, getMouseListeners, getMouseMotionListeners, getMousePosition, getMouseWheelListeners, getName, getParent, getPeer, getPropertyChangeListeners, getPropertyChangeListeners, getSize, getToolkit, getTreeLock, gotFocus, handleEvent, hasFocus, hide, imageUpdate, inside, isBackgroundSet, isCursorSet, isDisplayable, isEnabled, isFocusable, isFocusOwner, isFocusTraversable, isFontSet, isForegroundSet, isLightweight, isMaximumSizeSet, isMinimumSizeSet, isPreferredSizeSet, isShowing, isValid, isVisible, keyDown, keyUp, list, list, list, location, lostFocus, mouseDown, mouseDrag, mouseEnter, mouseExit, mouseMove, mouseUp, move, nextFocus, paintAll, postEvent, prepareImage, prepareImage, remove, removeComponentListener, removeFocusListener, removeHierarchyBoundsListener, removeHierarchyListener, removeInputMethodListener, removeKeyListener, removeMouseListener, removeMouseMotionListener, removeMouseWheelListener, removePropertyChangeListener, repaint, repaint, repaint, resize, resize, setBounds, setBounds, setComponentOrientation, setCursor, setDropTarget, setFocusable, setFocusTraversalKeysEnabled, setIgnoreRepaint, setLocale, setLocation, setLocation, setName, setSize, setSize, show, show, size, toString, transferFocus, transferFocusUpCycle`

**Methods inherited from class java.lang.Object**

`equals, getClass, hashCode, notify, notifyAll, wait, wait, wait`

**Constructor Detail**

### SetInstancesPanel

```
public SetInstancesPanel()
```

:   Create the panel.


**Method Detail**

### setParentFrame

```
public void setParentFrame(javax.swing.JFrame parent)
```

:   Sets the frame, this panel resides in. Used for displaying the close
    button, i.e., the close-button is visible if the given frame is not null.

    :   **Parameters:**: `parent` - the parent frame

---


### getParentFrame

```
public javax.swing.JFrame getParentFrame()
```

:   Returns the current frame the panel knows of, that it resides in. Can be
    null.

    :   **Returns:**: the current parent frame

---


### closeFrame

```
public void closeFrame()
```

:   closes the frame, i.e., the visibility is set to false

---


### setInstancesFromFileQ

```
public void setInstancesFromFileQ()
```

:   Queries the user for a file to load instances from, then loads the
    instances in a background process. This is done in the IO
    thread, and an error message is popped up if the IO thread is busy.

---


### setInstancesFromURLQ

```
public void setInstancesFromURLQ()
```

:   Queries the user for a URL to load instances from, then loads the
    instances in a background process. This is done in the IO
    thread, and an error message is popped up if the IO thread is busy.

---


### setInstances

```
public void setInstances(Instances i)
```

:   Updates the set of instances that is currently held by the panel

    :   **Parameters:**: `i` - a value of type 'Instances'

---


### getInstances

```
public Instances getInstances()
```

:   Gets the set of instances currently held by the panel

    :   **Returns:**: a value of type 'Instances'

---


### getLoader

```
public Loader getLoader()
```

:   Gets the currently used Loader

    :   **Returns:**: a value of type 'Loader'

---


### getSummary

```
public InstancesSummaryPanel getSummary()
```

:   Gets the instances summary panel associated with
    this panel

    :   **Returns:**: the instances summary panel

---


### setReadIncrementally

```
public void setReadIncrementally(boolean incremental)
```

:   Sets whether or not instances should be read incrementally
    by the Loader. If incremental reading is used, then
    the client should obtain the Loader object (by calling
    getLoader()) and read the instances one at a time. If
    batch loading is used, then SetInstancesPanel will load
    the data into memory inside of a separate thread and notify
    the client when the operation is complete. The client can
    then retrieve the instances by calling getInstances().

    :   **Parameters:**: `incremental` - true if instances are to be read incrementally

---


### getReadIncrementally

```
public boolean getReadIncrementally()
```

:   Gets whether instances are to be read incrementally or not

    :   **Returns:**: true if instances are to be read incrementally

---


### addPropertyChangeListener

```
public void addPropertyChangeListener(java.beans.PropertyChangeListener l)
```

:   Adds a PropertyChangeListener who will be notified of value changes.

    :   **Overrides:**: `addPropertyChangeListener` in class `java.awt.Container`
    :   **Parameters:**: `l` - a value of type 'PropertyChangeListener'

---


### removePropertyChangeListener

```
public void removePropertyChangeListener(java.beans.PropertyChangeListener l)
```

:   Removes a PropertyChangeListener.

    :   **Overrides:**: `removePropertyChangeListener` in class `java.awt.Component`
    :   **Parameters:**: `l` - a value of type 'PropertyChangeListener'


---


|  |  |  |
| --- | --- | --- |
| **Overview** \| **Package** \| **Class** \| **Tree** \| **Deprecated** \| **Index** \| **Help** \| **Weka's home** | |  |
| **PREV CLASS**   **NEXT CLASS** | **FRAMES**    **NO FRAMES**     **All Classes** |  |
| SUMMARY: NESTED \| FIELD \| CONSTR \| METHOD | DETAIL: FIELD \| CONSTR \| METHOD |  |


---
